# Ghosts of infections past: using archival samples to understand a century of monkeypox virus prevalence among host communities across space and time

**DOI:** 10.1098/rsos.171089

**Published:** 2018-01-31

**Authors:** Madeline S. Tiee, Ryan J. Harrigan, Henri A. Thomassen, Thomas B. Smith

**Affiliations:** 1 Department of Ecology and Evolutionary Biology, Institute of the Environment and Sustainability, University of California, Los Angeles, CA, USA; 2 Center for Tropical Research, Institute of the Environment and Sustainability, University of California, Los Angeles, CA, USA; 3 Institute of Evolution and Ecology, University of Tübingen, Tübingen, Germany

**Keywords:** monkeypox, zoonotic diseases, ancient DNA, museum specimens, *Funisciurus*, host community

## Abstract

Infectious diseases that originate from multiple wildlife hosts can be complex and problematic to manage. A full understanding is further limited by large temporal and spatial gaps in sampling. However, these limitations can be overcome, in part, by using historical samples, such as those derived from museum collections. Here, we screened over 1000 museum specimens collected over the past 120 years to examine the historical distribution and prevalence of monkeypox virus (MPXV) in five species of African rope squirrel (*Funisciurus* sp.) collected across Central Africa. We found evidence of MPXV infections in host species as early as 1899, half a century earlier than the first recognized case of MPXV in 1958, supporting the suggestion that historic pox-like outbreaks in humans and non-human primates may have been caused by MPXV rather than smallpox as originally thought. MPX viral DNA was found in 93 of 1038 (9.0%) specimens from five *Funisciurus* species (*F. anerythrus*, *F. carruthersi*, *F. congicus*, *F. lemniscatus* and *F. pyrropus*), of which *F. carruthersi* and *pyrropus* had not previously been identified as potential MPXV hosts. We additionally documented relative prevalence rates of infection in museum specimens of *Funisciurus* and examined the spatial and temporal distribution of MPXV in these potential host species across nearly a hundred years (1899–1993).

## 1. Introduction

Zoonotic diseases, or diseases that originate from non-human animal hosts, represent over 60% of the emerging infectious diseases that negatively impact human populations [1,2]. Of these, more than half infect multiple animal hosts [2–4]. For these multi-host pathogens, it is essential to differentiate between reservoir host species that enable persistence of the pathogens within a community as opposed to incidental hosts that do not play roles in disease maintenance [5]. Haydon *et al.* [5] further define reservoirs within multi-host communities as encompassing ‘one or more epidemiologically connected populations or environments in which the pathogen can be permanently maintained and from which infection is transmitted to the defined target population'. The recent outbreaks of Ebola virus disease and Middle-East respiratory syndrome coronavirus (MERS-CoV) exemplify how challenging it can be to identify the species that constitute these multi-host communities and their relative roles in transmitting disease within host communities and into human populations [5–8]. To best mitigate human risk, an understanding of these potentially complex host communities is needed across broad temporal and spatial scales.

Traditional methods used to answer questions about disease host communities generally focus on evidence from epidemiological associations linking host ecology to human disease patterns, serological or genetic tests of wildlife, phylogenetic analysis or intervention studies such as ring-fencing, culling or vector control measures [5]. Archival samples and ancient DNA expand on these techniques by increasing the spatial and temporal scope of studies while also providing samples for multiple host species [9,10]. Over the past decade, archival samples, mummified remains and permafrost-preserved corpses have provided historical evidence of infection for various pathogens ranging from viruses to bacteria including influenza, *Borrelia burgdorferi*, HIV, chytrid fungus and avian poxviruses [10–12]. As long as the assumptions inherent to these methods are acknowledged and when possible controlled for (such as sampling bias or the effects of specimen preparation and preservation methodology on DNA quality and amplification), museum sampling can be valuable for identifying potential host species and understanding broad geographical and temporal patterns of disease, especially in regions difficult to sample.

Monkeypox virus (MPXV) is a zoonotic pathogen for which such museum sampling could offer valuable insight into its multi-host community. Past studies on avipoxviruses (part of the same double-stranded DNA virus family *Poxviridae*) demonstrated the potential value of museum specimens in providing evidence of pox infection in wild passerine birds as far as a century ago [11,12]. MPXV causes clinical symptoms similar to smallpox in humans, infecting human populations throughout Central Africa, predominantly in the Democratic Republic of Congo (DRC) [13–16]. Since the late 1980s, human populations in Central Africa have seen an unprecedented 20-fold increase in MPXV infections [17]. MPXV is known to infect a broad range of hosts, including humans, non-human primates and rodents; however, the current knowledge of primary MPXV host species is limited. The first recorded description of MPXV occurred in 1958; however, reports of pox-like outbreaks occur as early as 1936 in non-human primates and may be attributed to MPXV instead of smallpox [18].

Non-human primates and humans can be incidental hosts of MPXV, while rodents, specifically rope squirrels (*Funisciurus*), sun squirrels (*Heliosciurus*), giant pouched rats (*Cricetomys*) and African dormice (*Graphiurus*), have been implicated as primary hosts in Central and Western Africa for maintaining MPXV primarily based on serological and viral DNA amplification data [14,16,19–26]. The clustered, self-limiting and sporadic transmission patterns of MPXV in human populations seem to indicate that a majority of human cases result from direct transmission from wildlife [14,27,28]. Previous studies using interview data from outbreaks in the 1980s suggest that 72% [29] to 78.1% [14] of all MPXV cases result from contact with wildlife. If these estimates are accurate, it becomes crucial to understand the prevalence of MPXV among various host species and, more importantly, how MPXV prevalence level interfaces with the role that each species plays in disease maintenance and transmission into human populations. It remains unknown which host species are required to maintain MPXV circulation within host communities, and whether these are different from the species that contribute to infections in human populations.

As a DNA virus, MPXV offers the unique opportunity for using museum specimens to gain perspective into the historical prevalence and distribution of a virus within host populations, as the genealogical remnants of an infection are likely to persist in the host long after death and/or museum preparation [10]. *Funisciurus*, when infected in laboratory experiments, develop skin lesions similar to those seen in other animal species and shed the infectious virus for 5 to 22 days. The presence of viral DNA in the skin could be indicative of active infections or a measure of infection in the past; little is understood about how long viral DNA persists in the skin after MPXV infection or the resolution of skin lesions. Until this study, all evidence of MPXV infection of the Congo-Basin strain in wild rodent populations, except for the singular isolation of MPXV virus in *F. anerythrus*, has historically been serological in nature, and from a limited time period [16,20,21,23,26].

We screened 1038 *Funisciurus* museum specimens spanning nearly a hundred years (1899–1993) from Central Africa for MPX viral DNA. The objectives of our study were to (i) investigate the potential for using museum skin specimens in identifying MPXV infection in host species, (ii) compare the prevalence levels of MPXV within various purported host species, (iii) examine the spatial and temporal patterns of MPXV prevalence and (iv) explore the impact of particular host communities and richness on MPXV prevalence.

## 2. Material and methods

### 2.1. Sample collection

*Funisciurus* skin samples were collected from dried specimens in 2012 from the Royal Museum for Central Africa (RMCA, *n* = 748 samples) in Tervuren, Belgium and in 2014 from the American Natural History Museum (AMNH, *n* = 329 samples) in New York City, USA. A 9–25 mm^2^ sample was collected from the ventral side of the proximal axillary front legs (armpit), lower neck or abdomen with an effort to preserve the appearance of each specimen. No obvious skin lesions were noted during sample collection. Samples were stored dry in collection tubes at room temperature prior to DNA extraction. Past laboratory experiments on the 2003 USA outbreak of MPXV within rodent populations showed that skin served as a reliable tissue for MPXV DNA detection [25]. Species identifications were assigned based on accession tags on each individual and cross-checked with the database of the museum.

### 2.2. Authenticity of positives

As suggested by Gilbert *et al.* [30], we designed our study to prevent contamination by using five of the nine criteria first recommended by Cooper and Poinar [31] for the authenticity of museum studies: (i) physically isolated work areas to keep DNA samples separated from PCR products, (ii) negative controls, (iii) appropriate molecular behaviour (e.g. longer amplicons should amplify less often than shorter ones), (iv) quantification by RT-PCR and (v) associated remains (e.g. ability to amplify squirrel DNA provides evidence of DNA quality). We did not use all nine recommended criteria because our study system fell into the low-risk category as established by Gilbert *et al.* (for more, see electronic supplementary material, methods section).

### 2.3. DNA extraction and quality assessment

DNA extraction was performed using a DNeasy® Blood and Tissue Kit (QIAGEN) according to the manufacturer's recommendations. DNA concentration and purity were assessed using a NanoDrop™ 2000 full-spectrum UV-vis spectrophotometer (Thermo Fisher Scientific). DNA extracts were stored at –20°C until testing was performed.

We used PCR amplification and gel visualization of the common vertebrate gene beta-actin from host DNA (associated remains) as a positive control to ensure DNA preservation quality and lack of PCR inhibition in each sample [10,32]. PCR reactions were repeated up to four times, and samples in which beta-actin could not be amplified were dropped from the study. In all, 39 samples were excluded, and a final dataset consisting of 1038 samples was screened for MPXV DNA from the following species: *F. anerythrus*, *bayoni*, *carruthersi*, *congicus*, *isabella*, *lemniscatus*, *leonis*, *leucogenys*, *pyrropus* and *substriatus* (table 1, electronic supplementary material, table S1).

**Table 1.** MPXV-positive samples by species and amplicon. Sample size for each species, number of positives and MPXV prevalence of positive samples for (i) G2R_G amplicon, (ii) G2R_WA amplicon or (iii) either amplicon with Clopper–Pearson exact 95% confidence intervals [33,34].

		no. positive			prevalence					
funisciurus species	no. tested	G2R_G	G2R_WA	either	G2R_G	95% CI	G2R_WA	95% CI	either	95% CI
*F. anerythrus*	362	12	38	45	0.033	0.017–0.057	0.10	0.075–0.14	0.12	0.092–0.16
*F. bayoni*	7	0	0	0	0	0–0.41	0	0–0.41	0	0–0.41
*F. carruthersi*	109	1	2	3	0.0092	0.00023–0.050	0.018	0.0022–0.065	0.028	0.0057–0.078
*F. congicus*	239	15	24	32	0.063	0.036–0.10	0.10	0.065–0.15	0.13	0.093–0.18
*F. isabella*	18	0	0	0	0	0–0.19	0	0–0.19	0	0–0.19
*F. lemniscatus*	82	2	3	5	0.024	0.0030–0.085	0.037	0.0076–0.10	0.061	0.020–0.14
*F. leonis*	2	0	0	0	0	0–0.84	0	0–0.84	0	0–0.84
*F. leucogenys*	6	0	0	0	0	0–0.46	0	0–0.46	0	0–0.46
*F. pyrropus*	201	5	5	8	0.025	0.0081–0.057	0.025	0.0081–0.057	0.040	0.017–0.077
*F. substriatus*	1	0	0	0	0	0–0.98	0	0–0.98	0	0–0.98
*F.* spp.	11	0	0	0	0	0–0.28	0	0–0.28	0	0–0.28
total	1038	35	72	93	0.034	0.024–0.047	0.069	0.055–0.087	0.090	0.073–0.11

To assess DNA quality and fragment length, and to ensure that positive samples were suitable for further MPXV screening, eight random samples were analysed using an Agilent 2100 Bioanalyzer® and Agilent High Sensitivity DNA kit (Agilent Technologies, Inc.). The average length of DNA in our samples was 218 bp (range = 29–1000 bp).

### 2.4. Monkeypox virus screening

Samples were screened for MPX viral DNA through a combination of (i) real-time PCR (RT-PCR), (ii) high-resolution melting (HRM) analysis and (iii) Sanger DNA sequencing to ensure the sensitivity and specific detection of MPXV. When coupled together, RT-PCR and HRM allow for the amplification and sensitive detection of DNA at low copy numbers [35]. Sanger sequencing was used to then verify HRM putative positives. Using a touchdown PCR protocol on a LightCycler® 480 Real-Time PCR System (Roche Diagnostics), RT-PCR assays were screened for two different MPXV amplicons G2R_G (123-bp) and G2R_WA (101 or 104-bp). Both these amplicons were developed by Li *et al.* [36] and represent different regions of the tumour necrosis factor receptor gene. We modified the primers used by Li *et al.* [36] to optimize RT-PCR amplification without the use of a probe. A single nucleotide polymorphism in G2R_G and a 3 bp deletion in the Congo-Basin strain in G2R_WA allowed for the distinction between the West African and Congo-Basin strains of MPXV [36]. Samples were loaded onto clear 384-well plates, with four replicates for each amplicon. Amplicons were run on separate plates with negative controls and, in the case of G2R_WA plates, serial dilutions of an altered positive control. The crossing point (Cp) for the G2R_G positive samples was 48.48 (95% CI, 47.11–49.84) and the Cp for the G2R_WA samples was 52.77 (95% CI, 52.17–53.37). For more details, see electronic supplementary material.

After the completion of PCR, HRM analysis was performed on all samples to confirm PCR specificity. Melt temperatures (*T*_m_) of RT-PCR products were determined through analysis of melt curves using Roche LightCycler® 480 Software version 1.5.039 (Roche Diagnostics) and confirmed with the *T*_m_ for serial dilutions of positive controls. Putative MPXV positives of the G2R_G amplicon melted on average at 79.6°C (95% CI, 79.37–79.83°C), and all G2R_WA amplicons melted at 78.89°C (95% CI, 78.66–79.13°C). Any *T*_m_ peaks at temperatures more than 0.5°C outside of the average melting temperature for each amplicon were attributed to non-specific amplification or primer-dimer. All RT-PCR products were stored at –20°C prior to sequencing and stored in a different room and freezer from DNA extracts.

All RT-PCR products from HRM putative positives were sent to Beckman Coulter Genomics (Danvers, MA) for product purification and Sanger DNA sequencing. Sequences were aligned to the G2R_G and G2R_WA amplicons using Geneious version 7.1.3 [37]. Samples were considered positive for MPXV DNA if they could be sequenced for either the G2R_G or G2R_WA amplicon for at least one of the four replicates. On average, each positive sample had 1.5 positive sequences (from two amplicons with four replicates). More specifically, 20.7% of positive samples had two positive sequences, 2.2% had three positive sequences and 7.6% had four positive sequences.

### 2.5. Statistical methods

#### 2.5.1. Comparing monkeypox virus infection across species, collection year and museum

Of the ten species screened, only five species (*F. anerythrus*, *carruthersi*, *congicus*, *lemniscatus* and *pyrropus*) that screened positive for MPXV infection were included in our statistical models. Using binomial logistical regression models and the R packages *stats* and *car* [33,38], we examined the effect of several variables on MPXV status (positive or negative). Variables were incorporated into the model as fixed effects and included ‘species', ‘5-year collection period' (collection year binned by 5-year period to avoid inflation of predictors), ‘collection month', ‘museum', ‘DNA quality' (NanoDrop 260/280 ratio), ‘DNA concentration' (ng/μl), ‘year of collection' (collection year as a continuous variable), ‘sex', ‘age group of specimen' (juvenile versus adult) and ‘geographical location' (grouped by administrative area). These variables were chosen to better understand what effect spatio-temporal, demographic and museum preservation factors might have on our ability to detect MPXV. All possible model permutations were compared by Akaike information criterion (AIC) values using the R package *glmulti* [39]. The best model was further simplified using backward elimination and likelihood-ratio chi-squared tests of nested models. Tukey's all pairwise difference tests were conducted on the simplified model to compare MPXV outcome between species, collection period and museums using the R package *multcomp* [40]. All statistical analyses, data manipulation and graphical plots were performed using RStudio [41], R [33], QGIS [42] and the *R* package *ggplot2* [33,43]. See electronic supplementary material for details.

#### 2.5.2. Comparing host community composition to monkeypox virus infection

To understand the effects of host community composition on MPXV infection, a mixed-effects Poisson regression model with a logit link was used to analyse MPXV-positive counts across geographical areas and compared to the presence/absence of various possible host species. Known species distributions for various *Funisciurus* and *Heliosciurus* species were obtained from the IUCN and used for these analyses [44]. Mixed-effects models were fit to data for each MPXV-positive species separately using the R package *lme4* [33,45] and controlled for variation between years, museums and sampling effort by including collection period and museum as random effects and an exposure variable. Model selection and averaging were done in the R package *MuMIn* [33,46]. See electronic supplementary material for details.

## 3. Results

Results suggest that the use of museum specimens could effectively reveal historical infections of MPXV across broad geographical scales and within multiple host specimens. We found evidence of MPXV circulating in host species as early as 1899, identified two new potential host species (*F. carruthersi* and *pyrropus*) and verified MPXV infections within *F. anerythrus*, *congicus* and *lemniscatus*.

**Figure 1.**
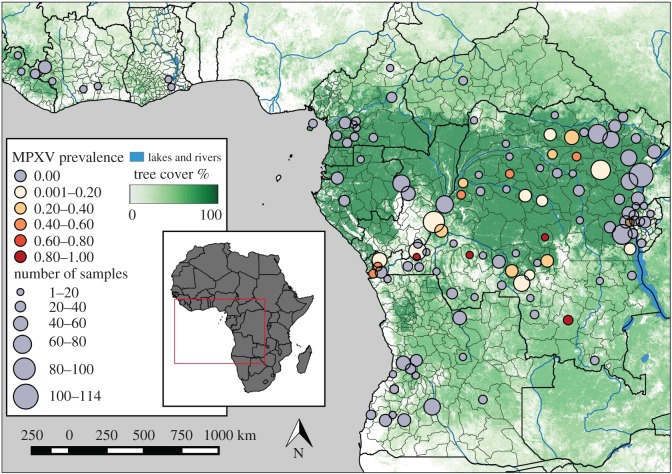
MPXV prevalence of all *Funisciurus* samples across local administrative areas. Prevalence levels for MPXV DNA are grouped by administrative areas for all museum skin samples. Dot size corresponds to the number of samples per area (*n* ≤ 114), while colour represents particular ranges of MPXV prevalence levels: grey for areas with no MPXV-positive samples, light reds for low MPXV prevalence and darker reds for high levels. Underlying layer represents tree cover, with darker greens corresponding to high percentages of cover [47,48]. Local administrative areas were geo-referenced to centroid coordinates.

**Figure 2.**
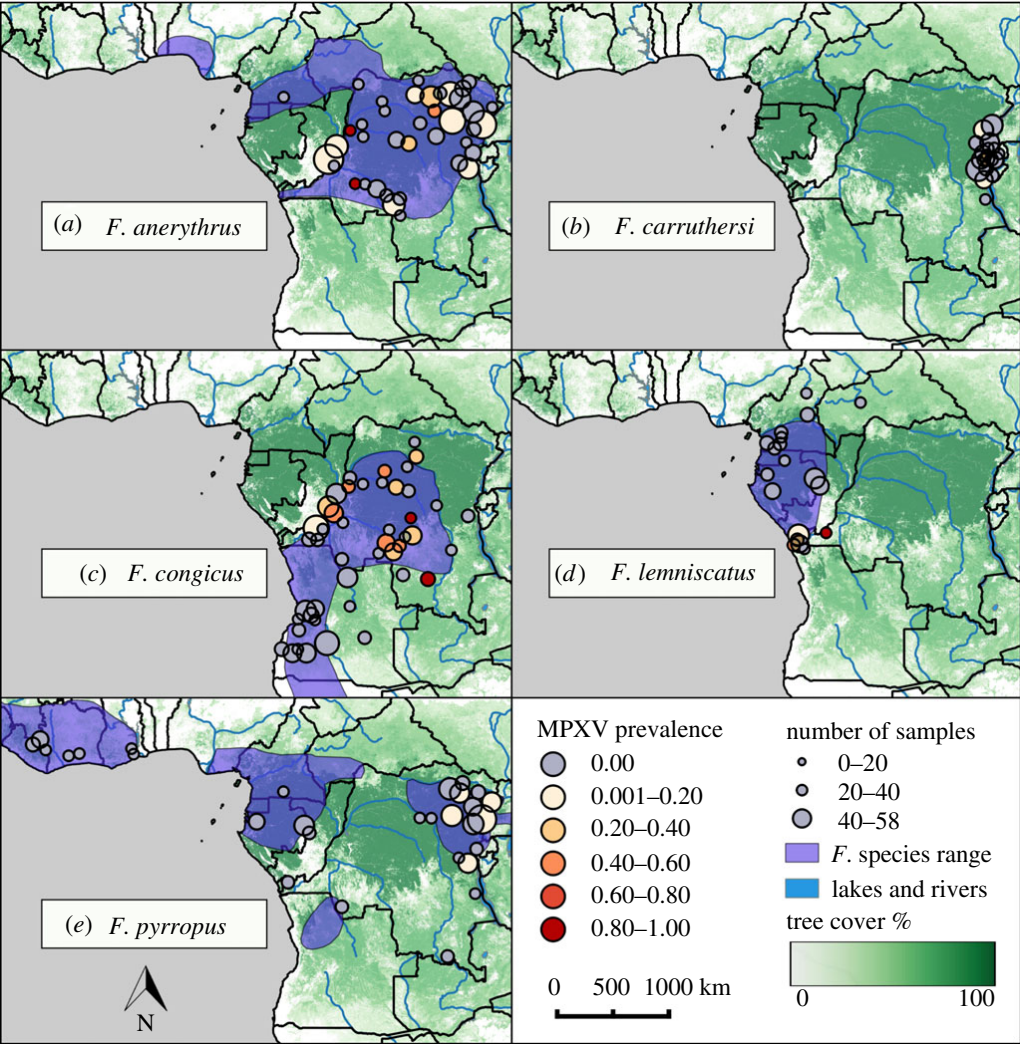
MPXV prevalence across local administrative areas for MPXV-positive species. MPXV prevalence levels are shown across administrative areas for MPXV-positives species: (*a*) *F. anerythrus*, (*b*) *F. carruthersi*, (*c*) *F. congicus*, (*d*) *F. lemniscatus* and (*e*) *F. pyrropus*. Prevalence levels are shown by dot coloration with areas with no MPXV-positive samples as grey dots, low prevalence as light reds and high prevalence as dark reds. Size of dots corresponds to the sampling number per locality (*n* ≤ 58). Estimated species ranges are shown in purple [44]. Underlying layer represents tree cover, with darker greens corresponding to high percentages of cover [47,48]. Local administrative areas were geo-referenced to centroid coordinates.

The overall MPXV prevalence level for either MPXV amplicon was 9.0% (93/1038, table 1). All positive samples belonged to the Congo-Basin strain of MPXV and were found in the DRC, except for one in the Central African Republic, with large numbers of positives occurring in the northwestern provinces of Kivu and Orientale, the southern province of Kasai-Occidental and the eastern provinces of Bandundu, Équateur and Bas-Congo (figures 1 and 2). The amplicons used were highly conserved and no genetic diversity was found between samples.

**Figure 3.**
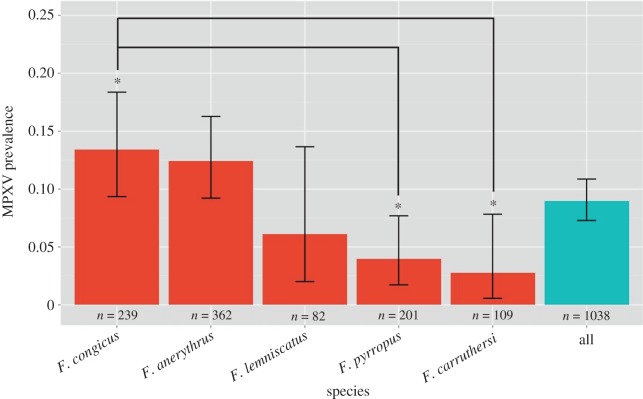
Comparison of MPXV prevalence among skin specimens by species. The total prevalence level for all sample specimens is shown along with prevalence levels for all positive species: *F. congicus*, *anerythrus*, *lemniscatus*, *pyrropus* and *carruthersi*. Error bars correspond to Clopper–Pearson exact 95% confidence intervals. Asterisks denote significant Tukey's pairwise comparisons (*p < 0.05) in MPXV prevalence levels between species. The results include all positives for both amplicons (G2R_G and G2R_WA) tested (for amplicon-specific results, see electronic supplementary material, figure S1 and table 1).

Of the ten species tested, five species (*F. anerythrus*, *carruthersi*, *congicus*, *lemniscatus*, *pyrropus*) had at least one individual positive for MPXV DNA. The prevalence of MPXV was 12% (45/362) for *F. anerythrus*, 2.8% (3/109) for *F. carruthersi*, 13% (32/239) for *F. congicus*, 6.1% (5/82) for *F. lemniscatus* and 4% (8/201) for *F. pyrropus* (figure 3, electronic supplementary material, figure S1 and table 1).

### 3.1. Differences in monkeypox virus prevalence by species, year of collection and museum

A comparison of AIC values from various binomial logistic regression models yielded a best model (AIC = 352.85) that included museum, species, month of collection, collection period, sex and age of specimen (electronic supplementary material, table S2). The variables of DNA quality, DNA concentration, age group of squirrel or administrative area on MPXV infection outcome were insignificant for predicting MPXV infection outcome. Using stepwise backward elimination, the final simplified model (AIC = 500.04) included museum, species and collection period as significant (*p* < 0.05) variables in predicting MPXV infection outcome (table 2). In comparison to *F. anerythrus* for the same year bin and museum, a negative coefficient for *carruthersi*, *lemniscatus* and *pyrropus* suggested that these three species were less likely to be positive for MPXV. *F. congicus*, however, had a positive coefficient, suggesting that a sample from this species had slightly higher odds of being positive when compared with *anerythrus* from the same museum and time period (table 2).

**Table 2.** Binomial logistic regression model of MPXV-positive/negative outcome data. The final simplified model included museum of origin, species and collection period as significant variables predicting MPXV infection outcome. Significance was determined by likelihood-ratio tests comparing models with and without a particular variable. Coefficients are given for the various variables. For museum, the coefficient is a comparison of the RMCA specimens with the AMNH, and for species, all species are compared with *F. anerythrus*.

variable	*LR χ^2^*	d.f.	*p*-value	*β* coefficient
museum	71.97	1	<2.2 × 10^−16^	
AMNH				—
RMCA				+4.42
species	17.64	4	0.0014	
*F. anerythrus*				—
*F. carruthersi*				−1.40
*F. congicus*				+0.51
*F. lemniscatus*				−0.45
*F. pyrropus*				−0.99
collection period	37.13	17	0.0032	

**Figure 4.**
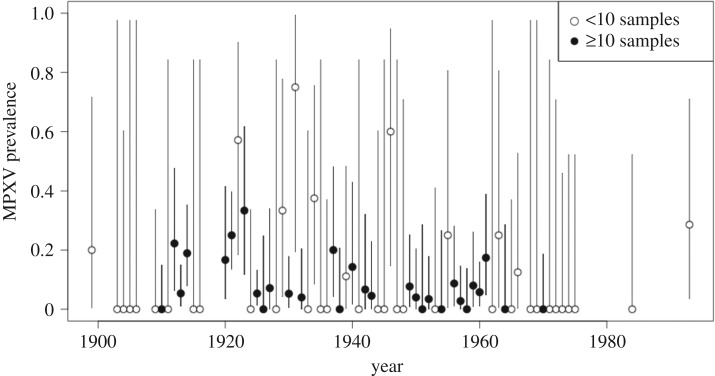
MPXV prevalence by year of collection for all species. MPXV prevalence by year of collection is shown for all species aggregated. All samples without a collection year were dropped from the dataset. Error bars correspond to Clopper–Pearson exact 95% confidence intervals [33,34]. To highlight years with larger sample sizes, black dots indicate years with 10 or more samples.

Tukey's all pairwise differences found a significant difference (*p* < 0.05) between the MPXV prevalence levels of *congicus* and *carruthersi* (*p = *0.028) and between *congicus* and *pyrropus* (*p *= 0.015) (figure 3). When comparing across collection periods while controlling for museum and species, MPXV-positive prevalence differed significantly between the collection period of 1921–1925 and 1956–1960 (*p* = 0.036) (figure 4). These differences were not in the expected direction; however, samples found over the periods of 1921–1925 had a higher odds of being positive when compared with the time period of 1956–1960. No general seasonal trends were seen by month of collection (electronic supplementary material, figure S2).

Museum of origin was significant for predicting MPXV outcome, with an 83x higher likelihood of being MPXV positive if the sample came from the RMCA (table 2). Likewise, Tukey's pairwise differences found this to be a significant difference (*p *= 1.63 × 10^−5^). This difference might exist because DNA extracted from RMCA samples may be of slightly higher DNA concentration and quality than the AMNH samples (see electronic supplementary material, methods).

### 3.2. Host community effects

Poisson mixed-effects models for all species did not reveal any significant factors related to host community richness or species presence/absence in determining MPXV-positive count when both museum and year bin were included as random effects.

A pattern of MPXV prevalence was evident across *F. congicus*' range: all MPXV-positive *congicus* were in DRC, even though half of the species' range occurs to the south and into Angola (figure 2). Unfortunately, all Angola specimens originated from the AMNH; the museum origin variable in models confounded efforts to understand host community effects for *congicus.* Additionally, *congicus* was 18.41 times more likely to be positive in geographical areas where it overlapped with *anerythrus* (see electronic supplementary material, results).

Co-occurrence of MPXV-positive samples across species during the same year and in the same locality was seen on four occasions. All four involved *F. anerythrus*: MPXV-positive *anerythrus* samples overlapping in locality with *F. congicus* positives at Kunungu (1921 and 1937) and at the Mission of St Joseph Luluabourg (1923). In addition, the town of Lima (1960) had co-occurrences of *F. anerythrus* and *lemniscatus* positives.

## 4. Discussion

Our study is the first to survey MPXV within host species using museum specimens. Our findings suggest there is great potential for using museum collections to retrospectively investigate the historical prevalence and distribution of DNA viruses within host species.

### 4.1. Potential of using museum skin specimens for surveying monkeypox virus prevalence

Our results suggest that museum sampling can offer invaluable information about past infections and potential current ones: the detection of MPX viral DNA in specimens has allowed us to identify two new potential hosts; verify MPXV infection in purported hosts; and compare disease prevalence within various species and across broad spatio-temporal time scales. We found evidence of MPXV circulating in host species as early as 1899, supporting the suggestion that pox-like outbreaks in humans and non-human primates prior to the first described MPXV case in 1958 could have historically been caused by MPXV instead of smallpox [18,13].

MPXV prevalence levels estimated from museum specimens should be interpreted with an appropriate amount of caution due to the common issues inherent to studies of museum samples. Museum studies can be subject to sampling bias with uneven coverage across geographical areas. Additionally, the process of field collection to museum storage often does not follow precautions for avoiding contamination between specimens; instruments used in preparation are not sterilized between specimens and specimens are often in close contact to others within museum storage cabinets. Given the lack of genetic diversity in the amplicons chosen for this study, it is difficult to identify potential contamination events. In our study, MPXV prevalence varied greatly based on the museum of origin. This could be due to inconsistencies between museums related to specimen preparation, preservation and sample collection, which may dramatically affect DNA extraction and amplification [10,49–51].

### 4.2. Monkeypox virus prevalence in multiple potential host species

Among the species found to harbour MPX infections, our analyses have revealed striking differences in MPXV prevalence. Both *F. anerythrus* and *congicus* showed high MPXV prevalence levels and also have the largest distributional ranges in the DRC, suggesting that they may play a dominant role in the transmission of MPXV within host communities, and to secondary hosts such as humans (figure 2). In addition to geographical distribution, observed differences in MPXV prevalence may be due to species-specific social behaviours that affect MPXV transmission rates. For instance, *F. congicus* is a highly social species that often lives in groups of up to four individuals [52]. In Gabon, *F. anerythrus* individuals are often seen in close pairs that travel together and allo-groom while sitting in contact. By contrast, *F. lemniscatus* individuals are often in groups, but usually maintain 5–20 m spacing, while *F. pyrropus* individuals are largely solitary [53]. These social behaviours may contribute to the high MPXV infection levels seen in *F*. *anerythrus* and low MPXV infection levels seen in *F. lemniscatus* and *pyrropus*. Other non-behavioural differences between species could also account for these prevalence differences including susceptibility of different species, viral titre levels, duration of infection and tissue tropism for viral replication. MPXV was detected in at least one animal for all species with higher sample sizes (greater than 18), raising the possibility that other host species may be identified with further testing.

### 4.3. Spatio-temporal patterns of monkeypox virus prevalence

Evidence of both temporal and spatial heterogeneity in MPXV infections in Central Africa was found in our study. Higher prevalence levels in the 1920s in comparison to the 1950s could be a true signal of temporal variation, or may be attributable to sampling bias and spatial heterogeneity of MPXV. Areas sampled in the 1920s do not overlap exactly with those sampled in the 1950s; and sampling in earlier years may have, by chance, included areas of higher MPXV prevalence than those sampled in later years.

One explanation for the observed spatial heterogeneity is the distribution of ideal squirrel habitat, including humid lowland evergreen tropical forests, degraded agricultural lands or palm oil plantations [21,22,53]. Palm oil trees (*Elaeis guineensis*) provide *Funisciurus* populations with a steady food source and allow squirrels to reach higher densities ranging from 440 to 500 squirrels per square kilometre [21,22,53]. Previous studies using ecological niche modelling methods predicted spatial distributions of MPXV similar to this study, and coincide with humid lowland evergreen tropical forests [54–56]. MPXV spatial heterogeneity probably results from a combination of environmental limitations and the spatial structure and distribution of host communities.

### 4.4. Role of host community in monkeypox virus transmission

For the species *F. congicus*, important questions remain as to the factors that limit MPXV distribution to the DRC, and that account for the low MPXV prevalence in Angola specimens. *F. congicus* is MPXV positive in areas of the DRC that coincide with the distribution of *anerythrus*; similarly, almost all MPXV positives found in this study for all *Funisciurus* species overlap in range with *anerythrus*. Likewise, all four co-occurrences of positive samples in the same locality and year included *anerythrus*. These observed patterns might be explained by several factors: (i) *F. anerythrus* is the primary reservoir species and allows for transmission to other species only when they co-occur with *anerythrus*, (ii) MPXV persistence depends primarily on a critical host density of any competent host species, but is limited in Angola where only *F. congicus* and *pyrropus* coexist, (iii) other environmental constraints, such as the transition from DRC's forest habitat to Angola's woody savannah [57], limit MPXV to the area occupied by *anerythrus*, (iv) sampling and specimen collection is biased towards areas that are part of *anerythrus*' range and (v) differences between museum specimen preparation/storage affect the ability to detect viral DNA. Additional sampling of museum specimens for both the DRC and Angola is needed to determine if the *F. congicus* MPXV levels are truly this different across their range.

Khodakevich *et al.* [21] suggested that *F. anerythrus* is the main reservoir host maintaining transmission of MPXV in Central Africa, but that other species such as *Heliosciurus rufobrachium* play important roles in transmission in areas where they co-occur with *anerythrus*. Our study investigated MPXV prevalence in *Funisciurus* sp. only; in reality, other genera could be playing major roles in transmission. Of note is the important distinction between reservoir hosts that are able to sustain MPXV transmission, and other incidental host species that are infected but cannot or do not maintain MPXV [5,6]. Unfortunately, this distinction is challenging to make given the difficulties in determining thresholds for persistence and susceptibility in host populations, and in estimating transmission rates between species [6]. Our study cannot specifically determine which species maintain the virus; however, certain characteristics of *F. anerythrus* make it a strong candidate. In addition to its pair-forming social behaviour, *F. anerythrus* is typically found in higher abundance than other *Funisciurus* species, and is thought to be better at colonizing areas due to its ability to swim and opportunistically forage in both arboreal and terrestrial habitats [53].

However, it is important to note that the systematics and population genetics of *Funisciurus* are not well resolved. Cryptic host species and migration between regions could play a role in virus distribution. This study used the species as identified and assigned by the museum collections; however, it is possible that cryptic species may lead to misidentification if based on morphology alone [58]. Further studies will be needed to elucidate the phylogeny of the host species and the specimens used in this study.

### 4.5. Need for further research and broader implications

*Funisciurus* and *Heliosciurus* generally occupy forest habitats, but can often become agricultural pests [21,22,53,59]. Many animal populations suffer ill-effects from hunting or agricultural disturbance, but *Funisciurus* populations actually may increase in abundance in areas with more degraded habitat [60]. In much of the Congo Basin, bushmeat remains a primary source for protein, and Central African rodents are increasingly harvested in the region with decreased availability of larger mammal populations [59,61–63]. As pressures from bushmeat hunting, deforestation and agricultural activity increase, the risk of MPXV is likely to also increase with elevated contact rates with infected host species through hunting and pest management.

Within this complex multi-host system, this study has identified new potential hosts of MPXV (*F. carruthersi* and *pyrropus*), verified MPXV infection in purported hosts (*F. anerythrus*, *congicus* and *lemniscatus)*, and quantified relative differences in MPXV prevalence between these hosts. Notably, our results suggest that museum sampling not only offers invaluable information about past infections but could also guide efforts to understand current and future outbreaks. Ongoing outbreaks, such as the recent 2016 MPXV outbreaks among humans in Central African Republic and among chimpanzees in Cameroon, emphasize the need for more research and sampling efforts to understand these complex systems. Our results suggest that future surveillance of MPXV within host populations should (i) target *F. anerythrus* and *congicus* across their spatial distribution, (ii) examine genetic diversity among host species to elucidate transmission dynamics and (iii) analyse contact rates between humans and squirrels to better understand *Funisciurus* contributions to human infection.

## Supplementary Material

Electronic Supplemental Material

## Supplementary Material

Tiee_etal-Figure S1

## Supplementary Material

Tiee_etal-Figure S2

## Supplementary Material

Tiee_etal-Figure S3

## Supplementary Material

Tiee_etal-Table S1

## Supplementary Material

Tiee_etal-Table S2

## Supplementary Material

R code for analyses

## Supplementary Material

Raw data

## Supplementary Material

Species comparison data

## Data accessibility

The datasets supporting this article have been uploaded as part of the electronic supplementary materials.
