# Supplementary material for: Ghosts of infections past: using archival samples to understand a century of monkeypox virus prevalence among host communities across space and time
Source: R Soc Open Sci. 2018 Jan 31;5(1):171089. doi: 10.1098/rsos.171089 (PMC5792900; doi:10.1098/rsos.171089)
Supplement: Electronic Supplemental Material [file rsos171089supp1.docx]

**SUPPLEMENTAL MATERIALS:**

**MATERIALS & METHODS:**

1. ***Sample collection***

In all, 1077 *Funisciurus* skin samples were collected: 377 *F. anerythrus*, 7 *bayoni*, 112 *carruthersi*, 252 *congicus*, 18 *isabella*, 84 *lemniscatus*, 2 *leonis*, 6 *leucogenys*, 207 *pyrropus*, 1 *substriatus*, and 11 *Funisciurus* specimens not identified to species level. A total of 748 samples were from the RMCA, and 329 were from the AMNH.

1. ***DNA Quality Assessment Using Beta-Actin***

The forward primer for the beta-actin amplicon was 5’–CCCTGAAGTACCCCATTGAA–3’ and the reverse primer was 5’–CTTGAAGGTCTCAAACATGATCT–3’ (Integrated DNA Technologies Inc., Coralville, Iowa, USA). All beta-actin PCR reactions were run using the QIAGEN Multiplex PCR Kit (Hilden, Germany) and contained 1.5 μl of template DNA, 1 μl of 1 μM primer for both forward and reverse primers, 5 μl of 2x QIAGEN Multiplex PCR Master Mix, 1 μl 5x Q-Solution, 0.4 μl of 10 mg/ml bovine serum albumin, and 1.1 μl of RNase-free water. PCR reactions were run on 96-well plates with one negative using 1.5 μl QIAGEN AE Elution buffer (Hilden, Germany) in place of template DNA. The reaction was run on a MJ Research PTC-200 Peltier Thermal Cycler (MJ Research Inc., Waltham, Massachusetts, USA) with thermal cycling conditions including initial denaturation for 15 mins at 95°C, followed by 11 cycles of 30 s denaturation at 94°C, 90 s annealing phase at 60°C, and 60 s extension at 72°C, then 31 cycles of 30 s denaturation at 94°C, 90 s annealing at 55°C, and 60 s extension at 72°C, followed by a final extension for 30 mins at 60°C. All PCR products were stored at -20°C. PCR products were run on a 2% agarose gel and visualized using SYBR Safe DNA gel stain (Invitrogen, Eugene, Oregon, USA).

1. ***MPXV Screening***

Both amplicons G2R_G and G2R_WA were from the conservative tumor necrosis factor (TNF) receptor gene located within the inverted terminal repeat (ITR) region. For the G2R_G amplicon, we used the same reverse primer designed by Li *et al.* (1): 5’–GCTATCACATAATCTGGAAGCGTA–3’. Primer3 version 4.0.0 (2,3) was used to create a forward primer for G2R_G: 5’–GGAAGAGATATAGCACCACATGC–3’. For the G2R_WA amplicon, we used a forward primer that was almost identical to that used in Li *et al.* (1) but was shifted by 1-bp for a forward primer of 5’–TCACACCGTCTCTTCCACAG–3’. Primer3 (2,3) was used to create the optimal reverse primer for G2R_WA: 5’–ACGATGTGTCGTTGACTGGA–3’ (Integrated DNA Technologies Inc., Coralville, Iowa, USA and ValueGene Inc., San Diego, California, USA). G2R_G contains one single nucleotide polymorphism and G2R_WA includes a 3-bp deletion in the Congo-Basin strain to allow the distinction between the West African and the Congo Basin strains. G2R_G is a 123-bp amplicon, and G2R_WA is a 101-bp amplicon with the Congo Basin Strain deletion or a 104-bp amplicon for the West African strain.

Four negatives using 1 μl of QIAGEN Elution AE buffer in place of template DNA were run on each plate. For the first amplicon G2R_G, no positive controls were used to prevent contamination. For the second amplicon G2R_WA, an altered positive control oligo was used (Integrated DNA Technologies Inc., Coralville, Iowa, USA). This positive control oligo had a 5-bp section in the middle altered in sequence to allow discrimination between positive control contamination and wild positives. Six serial dilutions of the positive control were placed on each plate for the G2R_WA amplicon at concentrations of 0.1nM, 0.01 nM, 1 pM, 0.1 pM, 0.01 pM, and 0.001 pM.

All real-time PCR reactions contained 1 μl of template DNA, 0.50 μl of 1 μM concentration of each primer, 5 μl of the Roche LightCycler 480 High Resolution Melting Master Mix 2x concentrated, 1.75 μl of 25 mM magnesium (II) chloride, and 2.25 μl of de-ionized water (Roche Diagnostics, Basel, Switzerland). A touchdown PCR protocol with thermal cycling conditions of an initial denaturation cycle of 10 min at 95°C followed by 60 cycles of 10 s denaturation at 95°C, 15 s annealing at 65 to 53°C, and 10 s extension at 72°C. The annealing temperature began at 65°C and decreased with each cycle by 0.5°C until reaching a final annealing temperature of 53°C which was held for the remainder of cycles.

The high-resolution melting cycle included 60 s at 95°C, 60 s at 40°C, 1 s at 65°C, and then heating to 97°C with a ramp rate of 0.02°C/s.

1. ***Authenticity of Positives***

To prevent contamination, Cooper and Poinar (4) recommended nine criteria for the authenticity of museum studies. As suggested by Gilbert *et al.* (5), we designed our study to avoid contamination but did not use all recommended nine criteria because our study system falls into a low-risk category. The low-risk category includes studies that do not investigate ancient human DNA, paleopathogens and microorganisms for which modern counterparts are ubiquitous, or domestic plants/animals (5). As MPXV is a non-ubiquitous pathogen, we implemented five of the nine criteria: (1) physically isolated work areas, (2) negative controls, (3) appropriate molecular behavior, (4) quantification by RT-PCR, and (5) associated remains.

All PCR products and positive controls were stored in a room and freezer separate from where DNA extractions and RT-PCR plate preparation occurred. All plates were run with four negative controls: no negative controls came up positive during the course of the study. All positive controls used modified sequences to allow differentiation of positive control contamination from actual positives. Additionally, the use of Sanger sequencing as a final verification step ensured that any wild contamination that happened at low concentrations would sequence poorly. A comparison of putative HRM positives to Sanger-sequence verified positives shows that HRM was far more sensitive to DNA amplification at low copy numbers than Sanger sequencing (Figure S3). Furthermore, we amplified viral DNA of short length (<500 bp) and expected our shorter amplicon G2R_WA to amplify more often than our longer amplicon G2R_G (4,6). Additionally, we were able to quantify all our reactions using RT-PCR. All reactions amplified late in the cycles, however this is normal for museum studies, especially involving the amplification of something in low copy number like that of viral DNA within more concentrated host DNA (7). Our “associated remains” included using host DNA quality and amplification of the common gene beta-actin as a proxy for MPXV viral DNA quality (7).

1. ***Statistical methods: Administrative areas and spatial aggregation***

Samples were binned by year and location to increase sample size and allow for analyses that included spatial or temporal factors. Except for the interval of 1899-1905, collection year was binned into five-year periods: 1906-1910, 1911-1915, etc. Analyses were repeated across various year bins to ensure that binning method did not have an effect on model performance.

All sample localities were geo-referenced to similarly-sized administrative areas in order to include a measure of spatial heterogeneity and make comparisons within the same spatial resolution. Most museum specimen samples were geo-referenced to town level, however some were geo-referenced to the sous-préfecture, district, province, or country level. All localities for museum specimens were verified or found using the National Geospatial-Intelligence Agency’s GEOnet Names Server database (8) and Google Earth Landsat (Google, Landsat, SIO, NOAA, US Navy, NGA, GEBCO 2015). Due to differences in localities’ spatial extents, samples were analyzed at a lower spatial resolution and assigned to an administrative area. These administrative areas differed in level between countries but were chosen based on similarity in size: sous-préfecture (DRC), préfecture (Rwanda), município (Angola), district (Ghana and Republic of Congo), département (Cameroon, Cote d’Ivoire, and Gabon), province (Burundi and Equatorial Guinea), and county (Liberia) levels. All samples that could not be referenced to an administrative area or with spatial extents greater than these administrative areas were dropped from this analysis. For spatial comparisons of MPXV prevalence and positive counts across year bins and for various host-community richness indices, museum samples were aggregated by these administrative areas.

1. ***Statistics methods: Host community indices***

Potential host distributions were obtained from the IUCN *Red List of Threatened Species Version 2012.1* (9) for genera *Funisciurus* and *Heliosciurus*: *F. anerythrus*, *F. bayoni*, *F. carruthersi*, *F. congicus*, *F. isabella*, *F. lemniscatus*, *F. leonis*, *F. leucogenys*, *F. pyrropus*, and *H. gambianus*, *H. rufobrachium*, and *H. ruwenzorii*. Presence/absence of a species was coded as a binary variable at a 1-km spatial resolution and averaged across administrative areas. Additionally, various combinations of host community richness were calculated. Other indices of richness included the species richness of (i) genus *Funisciurus*, (ii) genus *Heliosciurus*, (iii) all species belonging to both genera, (iv) the presence of both *F. anerythrus* and *F. congicus*, (v) host species found to be MPXV positive in this study (*F. anerythrus*, *carruthersi*, *congicus*, *lemniscatus*, and *pyrropus*), and (vi) host richness found seropositive historically and PCR-positive in this study (*F. anerythrus*, *carruthersi*, *congicus*, *isabella*, *lemniscatus*, *pyrropus*, *gambianus*, *rufobrachium*). All spatial resampling, extraction of raster values, creation of species richness indices were performed using a combination of QGIS Version 2.6.0-Brighton (10), RStudio version 0.98.1091 (11), R version 3.0.2 (12), R package raster version 2.3-12 (13), and R package rgdal version 0.9-1 (14).

MPXV positive counts per administrative area were compared to the presence/absence of specific species and other indices of species richness using a mixed effects Poisson regression model with a logit link. Models were created for each MPXV-positive species separately and controlled for variation between years, museums, and sampling effort by including collection period and museum as random effects and an exposure variable. The exposure variable offset the model by a logarithmic function of the total number of samples from each administrative area, accounting for differences in opportunity to detect an MPXV positive due to sample size. All mixed effects models were fit to the data using R package lme4 (12,15). Model selection and averaging were done through R package MuMIn (12,16). Individual model variables were tested for significance using likelihood-ratio chi-squared tests, and overall model fit was measured through pseudo R-squared values and F-test. Residual deviance was compared to residual degrees of freedom to ensure that the data did not exhibit overdispersion.

**RESULTS:**

The overall MPXV prevalence level for either MPXV amplicon was 9.0% (93/1038, Table 1). When broken down by amplicon, the prevalence rates for MPXV DNA were overall 3.4% (35/1038) for the G2R_G amplicon and 6.9% (72/1038) for the G2R_WA amplicon (Figure S1, Table 1).

**(a) *Museum collection differences***

The significant difference in MPXV outcome seen in specimens based on museum of origin was further explored. The geographic distribution of samples varied widely by museum and overlap of RMCA and AMNH samples occurred primarily in the DRC. To ensure that this museum effect was not confounded by spatial representation, the data was constrained to the administrative areas where museums overlap. A mixed effects Poisson regression model fit to MPXV counts with collection year bin as a random effect and museum as a fixed variable was significant in *F. anerythrus* (*p*=0.0015, *LR χ^2^* =10.05) and *F. congicus* (*p*=0.046, *LR χ^2^* =3.97). Both saw a higher likelihood of MPXV positives for the RMCA specimens. Additionally, the concentration of DNA extracted from specimens was correlated to the museum origin. In a significant linear regression model fitted to the concentration of DNA, DNA concentration of the RMCA was 15.504 ng/μl (*p*=0.082, *SE =* 5.9, *n* = 945) higher than that of the AMNH specimens. Likewise, a linear model examining the relationship of DNA quality (260/280 ratio from the Nanodrop) showed a slightly significant association with slightly higher quality in the RMCA samples (*β* = 0.045, *p*=0.052, *SE* = 0.023, *n* = 945)).

**(b) *Host community results***

When museum was not included as a random effect in the mixed effects Poisson regression model, the presence/absence of *anerythrus* was significantly correlated to MPXV positive counts for *congicus*. 10 of the 11 top models using various species richness indices when ranked by AIC value included the presence/absence of *anerythrus.* Additionally, the top ranked model included the presence/absence of *anerythrus* as its only independent fixed variable. In this top model, the overall model was significant (*R^2^*=0.4306, *p*=7.6E-5, *F-value* =16, *df* = 1, 57) and the fixed variable of *anerythrus* presence/absence was significant in the model (*p*=3.41E-6, *LR χ^2^* =21.57, *df* = 1). The positive coefficient of 2.91 (*SE*= 0.86, *z-value* = 3.93, *p=*0.00069) for the *anerythrus* presence/absence variable suggests that the presence of *anerythrus* in the same administration area results in an 18.41 fold increase of MPXV positives in *congicus*. This highlights the pattern seen in MPXV prevalence across *congicus’* range: All MPXV-positive counts of *congicus* were found in the DRC (overlap with *anerythrus*) whereas all Angola *congicus* samples were MPXV-negative (no overlap with *anerythrus*). The reverse effect of the presence/absence *congicus* on *anerythrus* was not seen; the model using *congicus* co-occurrence as the explanatory variable did not significantly predict *anerythrus* MPXV positive counts (*p*=0.93, *LR χ^2^* =0.0086, *df* = 1). This may underscore the importance of *anerythrus* within interspecific transmission in MPXV dynamics or it may also be indicative of underlying environmental or host constraints that may limit what host-environment is suitable for MPXV survival to the range of *anerythrus*. Unfortunately all Angola samples originated from the AMNH, and it was hard to determine if this lack of positives in this part of *congicus’* range was due to an actual geographical difference in prevalence levels or a museum effect.

**DISCUSSION**

***Potential of using museum skin specimens for surveying MPXV prevalence***

Museum collections offer valuable resources for the study of disease, however prevalence levels estimated from the use of museum samples should be interpreted with caution. Museum studies often fall victim to sampling bias and suffer from limitations in DNA amplification, spatial resolution/coverage, and contamination prevention. Uneven sampling effort was evident in our study; samples appear to originate from locations that were more accessible in the field, and 83.1% (789/949) of our DRC samples were collected prior to the independence of Belgian Congo in 1961 when governmental status and stability affected collecting surveys. Unfortunately, sampling coverage in more recent years is poor, likely due to political unrest and dangerous field conditions, making it difficult to examine temporal changes in MPXV infection in host species within the context of smallpox eradication and waning smallpox vaccination cross-immunity.

In our study, MPXV prevalence varied greatly based on the museum of origin. This could be due to inconsistencies between museums related to specimen preparation, preservation, and sample collection which may dramatically affect DNA extraction and amplification (7,17,6,18). Museum collectors and museums used various and sometimes unsystematic methods for specimen preparation, preservation, and storage over the years, including chemical treatments with arsenic that may inhibit both the enzymes used in DNA extraction and PCR (17).

Other factors may have also influenced the MPXV screening in specimens and prevalence levels. Skin tissue has proved to be fairly useful for DNA amplification for various animals infected by the West African strain of MPXV (7), however viral DNA may be differentially sequestered in the host body depending on the species, transmission pathway, specific immunological host response, or viral targeting of host cells, especially in orthopoxviruses generally characterized by various forms of generalized vesiculopustular rashes and skin lesions (19). Furthermore, little is understood about how long viral DNA persists in the skin after MPXV infection; the presence of viral DNA within the skin may be indicative of active infections or could be a measure of past infection. In an acute infection such as MPXV, it is unlikely that our high prevalence levels represent active infections. Additionally, our samples were not always taken from the same parts of the skin and this may affect ability to detect MPXV.

Our study is also limited by the current genetic knowledge of MPXV. The MPXV amplicons created by Li *et al.* (1) are based on all available human orthopoxvirus genomes, presenting the possibility that the amplified region may actually be more diverse within host species and our primers may not efficiently anneal to areas with disparities in sequence. If this is the case, we could be systematically missing strains that vary across space for which there are no human isolates. For example, our lack of positives in Angola may be attributed to the lack of human isolates for these areas and a systematic bias in our method of detection. Finally, our conservative measure of verifying positives through sequencing means that we could be missing many putative positives. In general, the low copy number of pathogen DNA in comparison to host DNA complicates the process of amplifying MPXV DNA (7).

We have taken multiple measures to avoid contamination; however there is the possibility that some positives in our study result from wild or PCR contamination. Following five of Cooper and Poinar’s “nine criteria of authenticity” (4,5) does not exclude the possibility of cross-contamination from museum specimens while in storage prior to analyses (7). However the detection of wild contamination is unlikely as the low copy number of viral DNA will be further diluted, making it more difficult to PCR amplify and also sequence these contaminants (Figure S3). As expected, the amplification of G2R_WA (101-bp) resulted in higher MPXV prevalence rates because its length was shorter than the other amplicon G2R_F (123-bp). Our Bioanalyzer results estimated that the concentration of a 100-bp amplicon was about 6.2% higher than that of a 125-bp amplicon. The ratio of viral DNA to host DNA likely further exacerbates this concentration difference between amplicons, resulting in a further gap in MPXV detection due to low copy number. Furthermore, stochastic effects in real-time PCR reactions are observed when less than 10 copies of the target DNA are present in the reaction (20) and could vary dramatically depending on infection level or state of animal at death. Estimates of MPXV prevalence in skin specimens could be higher than seen if any contamination did occur or lower than in reality if our detection method was not sensitive enough.

SUPPLEMENTAL REFERENCES

1. Li Y, Zhao H, Wilkins K, Hughes C, Damon IK. Real-time PCR assays for the specific detection of monkeypox virus West African and Congo Basin strain DNA. J Virol Methods. 2010;169:223–7.

2. Untergasser A, Cutcutache I, Koressaar T, Ye J, Faircloth BC, Remm M, et al. Primer3-new capabilities and interfaces. Nucleic Acids Res. 2012;40(15):1–12.

3. Koressaar T, Remm M. Enhancements and modifications of primer design program Primer3. Bioinformatics. 2007;23(10):1289–91.

4. Cooper A, Poinar HN. Ancient DNA: Do It Right or Not at All. Sci Lett. 2000;

5. Gilbert MTP, Bandelt H-J, Hofreiter M, Barnes I. Assessing ancient DNA studies. Trends Ecol Evol. 2005;20(10):541–4.

6. Paabo S, Higuchi RG, Wilson AC. Ancient DNA and the Polymerase Chain Reaction: The Emerging Field of Molecular Archaeology. J Biol Chem. 1989;264(17):9709–12.

7. Tsangaras K, Greenwood AD. Museums and disease: using tissue archive and museum samples to study pathogens. Ann Anat. Elsevier GmbH.; 2012;194(1):58–73.

8. National Geospatial-Intelligence Agency. GEOnet Names Server database [Internet]. 2015 [cited 2015 Jan 1]. Available from: http://geonames.nga.mil/namesgaz/

9. IUCN. IUCN Red List of Threatened Species [Internet]. 2012. Report No.: 2012.2. Available from: http://www.iucnredlist.org

10. QGIS Development Team. QGIS Geographic Information System [Internet]. 2014. Available from: http://qgis.osgeo.org

11. RStudio. RStudio: Integrated development environment for R [Internet]. Boston, MA; 2012. Available from: http://www.rstudio.org

12. R Core Team. R: A language and environment for statistical computing. Vienna, Austria: R Foundation for Statistical Computing; 2013.

13. Hijmans RJ. raster: Geographic data analysis and modeling [Internet]. 2014. Available from: http://cran.r-project.org/package=raster

14. Bivand R, Keitt T, Rowlingson B. rgdal: Bindings for the Geospatial Data Abstraction Library [Internet]. 2014. Available from: http://cran.r-project.org/package=rgdal

15. Bates D, Maechler M, Bolker B, Walker S. lme4: Linear mixed-effects models using Eigen and S4 [Internet]. 2014. Available from: http://cran.r-project.org/package=lme4/

16. Bartoń K. MuMIn: Multi-Model Inference [Internet]. 2015. Available from: http://cran.r-project.org/package=MuMIn

17. Hall LM, Willcox MS, Jones DS. Association of enzyme inhibition with methods of museum skin preparation. Biotechniques. 1997;22(5):928–34.

18. Hofreiter M, Serre D, Poinar HN, Kuch M, Pääbo S. Ancient DNA. Nat Rev Genet. 2001;2(5):353–9.

19. Chapman JL, Nichols DK, Martinez MJ, Raymond JW. Animal models of orthopoxvirus infection. Vet Pathol. 2010;47(5):852–70.

20. Morrison TB, Weis JJ, Wittwer CT. Quantification of lowcopy transcripts by continuous SYBR® green I monitoring during amplification. Vol. 24, BioTechniques. 1998. p. 954–62.

21. Zhou L, Palais R a., Paxton CN, Geiersbach KB, Wittwer CT. Copy Number Assessment by Competitive PCR with Limiting Deoxynucleotide Triphosphates and High-Resolution Melting. Clin Chem [Internet]. 2015;61(5):724–33. Available from: http://www.clinchem.org/cgi/doi/10.1373/clinchem.2014.236208

**SUPPLEMENTAL FIGURE AND TABLE CAPTIONS**

**Figure S1. Comparison of MPXV prevalence among skin specimens for both amplicons**

MPXV prevalence levels and their Clopper-Pearson exact 95% confidence intervals are shown for the (i) G2R_G amplicon (red), (ii) G2R_WA amplicon (green), and (iii) either amplicon (blue). MPXV prevalence levels are given overall and for the five MPXV positive species: *Funisciurus anerythrus*, *F. carruthersi*, *F. congicus*, *F. lemniscatus*, and *F. pyrropus*.

**Figure S2. MPXV Prevalence by month of collection for all years and species**

MPXV prevalence rates are given by month of collection for all years and species. All samples without a collection month were dropped from the dataset. Error bars are the Clopper-Pearson exact 95% confidence intervals.

**Figure S3.** **Comparison of Sanger sequencing verification results to fluorescence levels of HRM putative positives**

Rate of fluorescence or the intensity of the T_m_ peak as calculated through analysis of HRM melting curves acts as a proxy for copy number of PCR product (21). Black circles correspond to the G2R_G amplicon and red points to the G2R_WA amplicon. This figure compares the sensitivity of HRM to that of Sanger sequencing. HRM putative positives melting in the correct melting range are more likely to be verified by Sanger-sequencing if the copy number is higher. This restricts the positives to those that occur in higher copy number, avoiding wild contamination. An ANOVA rejects the null hypothesis that the mean T_m_ peak intensity of fluorescence for Sanger-sequenced positives is equal to the mean for Sanger-sequenced negatives (*p* < 2.2E-16, *F* = 190.7, *df* = 1).

**Table S1. Number of *Funisciurus* samples screened for MPX viral DNA based on country of origin and species**

**Table S2. Binomial logistic regression model analysis of MPXV positive/negative outcome data based on AIC value**

The best model based on AIC value (AIC = 352.85) included museum of origin, species, month of collection, collection period, sex, and age of specimen as significant variables predicting MPXV infection outcome. Significance was determined by likelihood ratio tests comparing the model with and without a particular variable
