# Supplementary material for: Ghosts of infections past: using archival samples to understand a century of monkeypox virus prevalence among host communities across space and time
Source: R Soc Open Sci. 2018 Jan 31;5(1):171089. doi: 10.1098/rsos.171089 (PMC5792900; doi:10.1098/rsos.171089)
Supplement: Tiee_etal-Figure S1 [file rsos171089supp2.pdf]

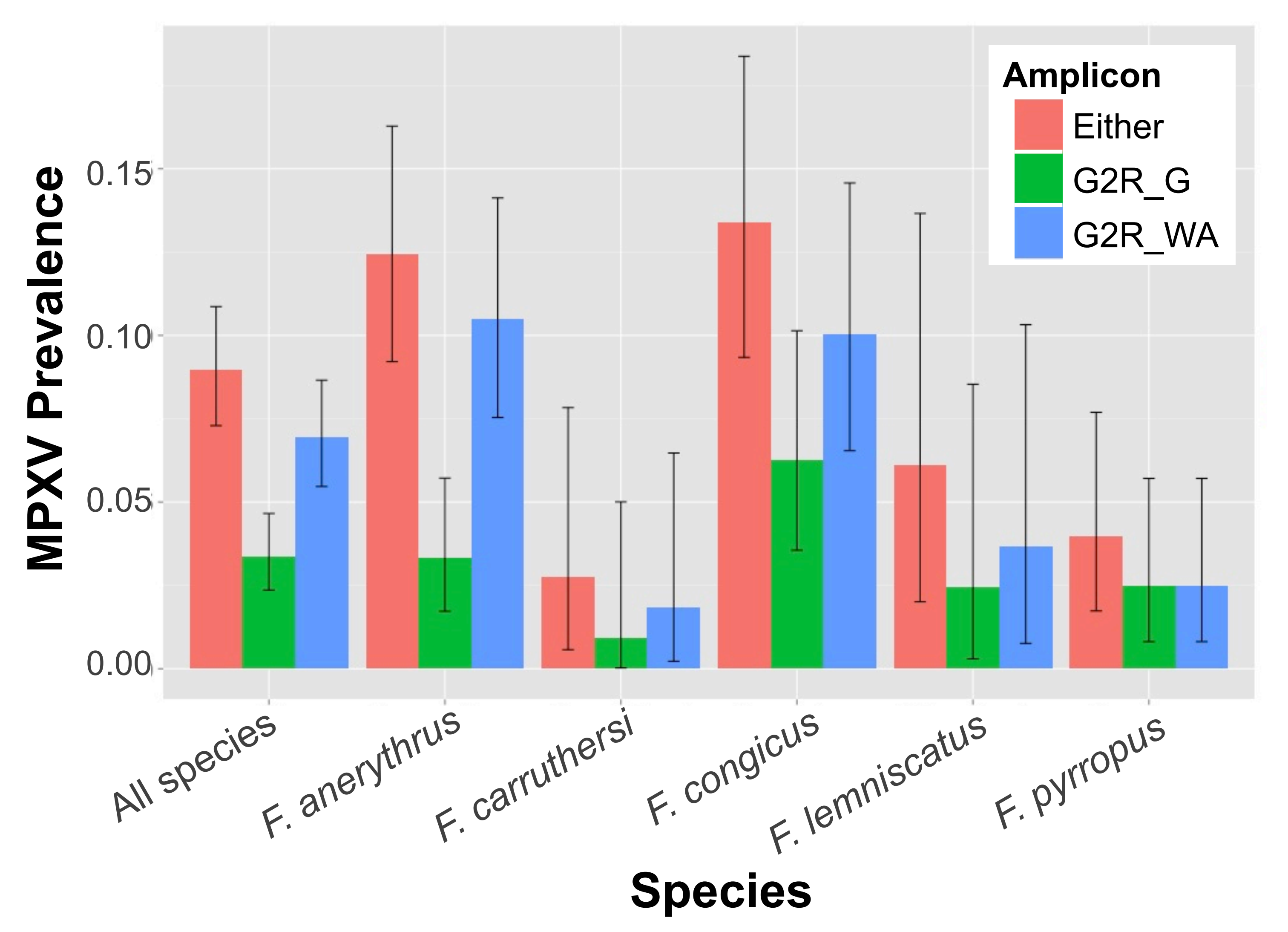

Amplicon
Either
G2R_G
G2R_WA
MPXV Prevalence
0.15
0.10
0.05
0.00
All species
F. anerythrus
F. carruthersi
F. congicus
F. lemniscatus
F. pyrropus
Species
