# Supplementary figures and images for: Ghosts of infections past: using archival samples to understand a century of monkeypox virus prevalence among host communities across space and time
Source: R Soc Open Sci. 2018 Jan 31;5(1):171089. doi: 10.1098/rsos.171089 (PMC5792900; doi:10.1098/rsos.171089)

**MPXV Prevalence**

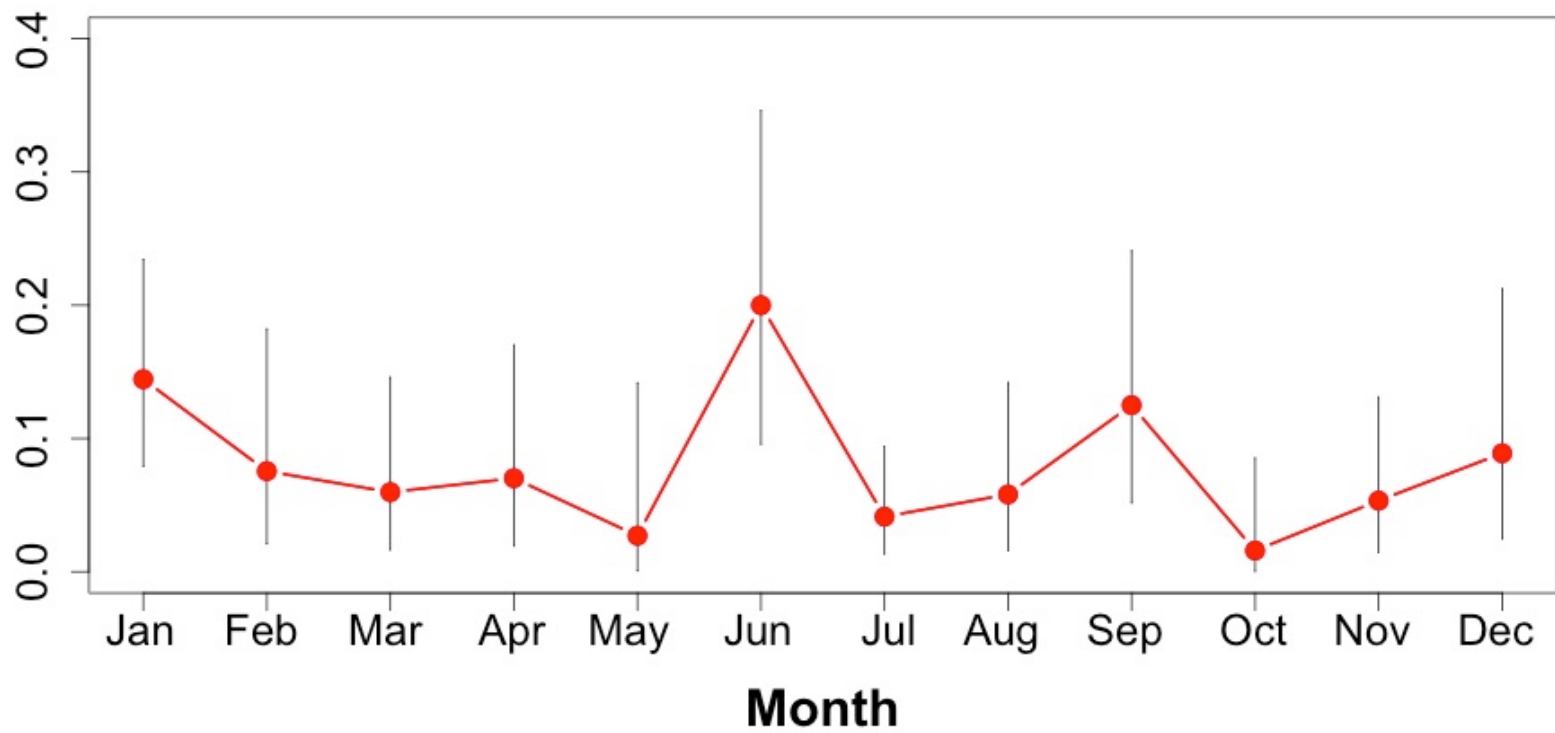

Supplement: Tiee_etal-Figure S2 [file rsos171089supp3.pdf]

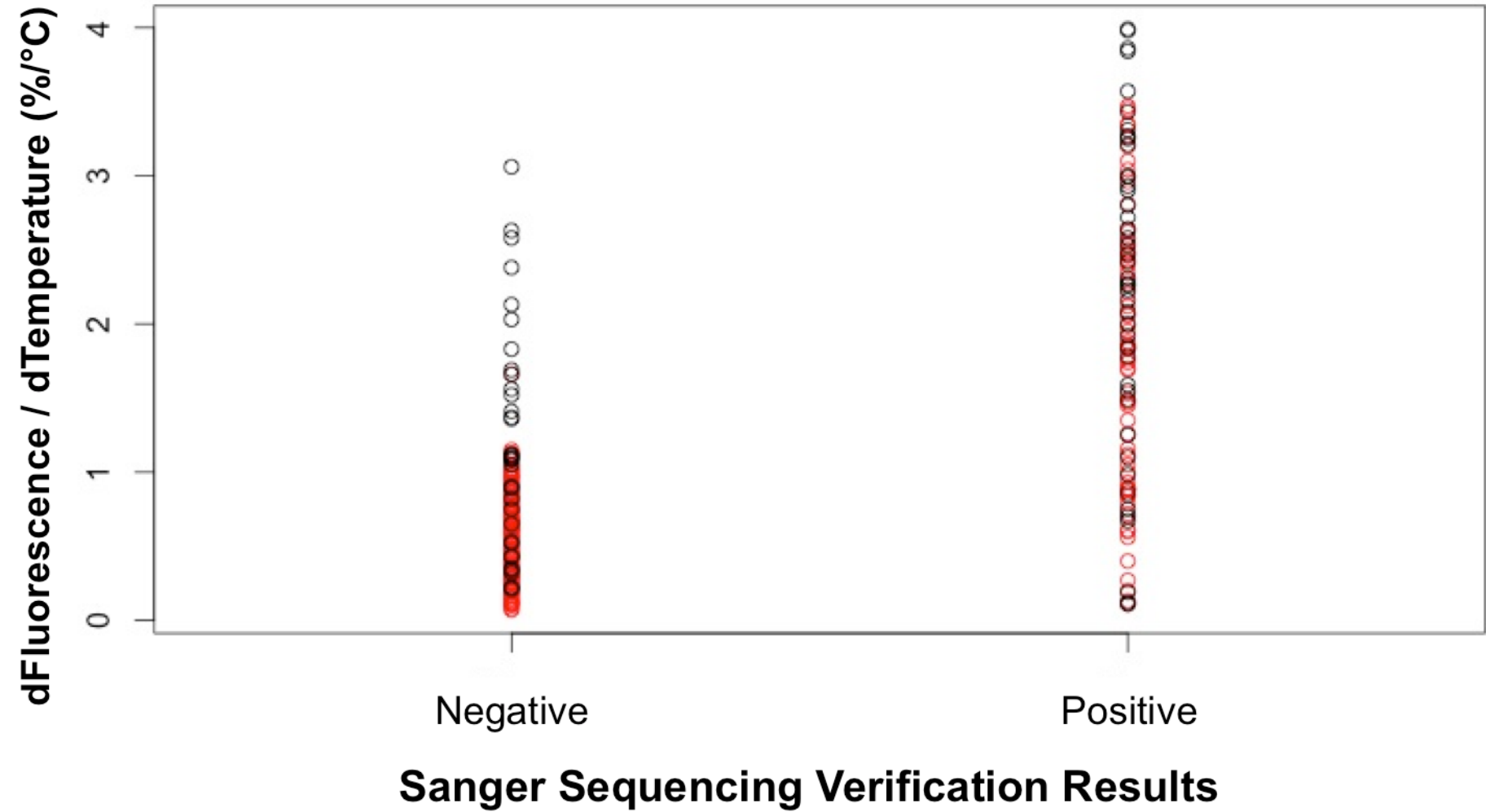

Supplement: Tiee_etal-Figure S3 [file rsos171089supp4.pdf]
