# Supplementary material for: Ghosts of infections past: using archival samples to understand a century of monkeypox virus prevalence among host communities across space and time
Source: R Soc Open Sci. 2018 Jan 31;5(1):171089. doi: 10.1098/rsos.171089 (PMC5792900; doi:10.1098/rsos.171089)
Supplement: Tiee_etal-Table S1 [file rsos171089supp5.pdf]

COUNTRY OF ORIGIN	Number of *Funisciurus* Samples Screened for MPX Viral DNA											
	anerythrus	*bayoni*	*carruthersi*	*congicus*	*isabella*	*lemniscatus*	*leonis*	*leucogenys*	*pyrropus*	*substriatus*	Unknown *spp.*	All species
Angola	---	---	---	92	---	---	---	---	---	---	---	92
Burundi	---	---	3	---	---	---	---	---	---	---	---	3
Cameroon	1	---	---	---	6	14	---	---	1	4	---	26
Central African Rep.	5	---	---	---	---	1	---	---	1	1	---	8
Congo, Republic of	14	---	---	5	10	25	---	---	14	1	---	69
Democratic Republic of Congo (DRC)	331	7	92	128	---	29	---	---	159	1	---	747
Equatorial Guinea	---	---	---	---	---	---	---	2	---	---	---	2
Gabon	---	---	---	---	1	9	---	---	5	---	---	15
Ghana	---	---	---	---	---	---	---	---	2	---	1	3
Cote d'Ivoire	---	---	---	---	---	---	---	4	9	---	---	13
Liberia	---	---	---	---	---	---	2	---	---	---	---	2
Rwanda	---	---	13	---	---	---	---	---	---	---	---	13
Uganda	---	---	1	---	---	---	---	---	---	---	---	1
Unknown	11	---	---	14	1	4	---	---	10	4	---	44
**TOTAL**	**362**	**7**	**109**	**239**	**18**	**82**	**2**	**6**	**201**	**11**	**1**	**1038**
