# Supplementary material for: Ghosts of infections past: using archival samples to understand a century of monkeypox virus prevalence among host communities across space and time
Source: R Soc Open Sci. 2018 Jan 31;5(1):171089. doi: 10.1098/rsos.171089 (PMC5792900; doi:10.1098/rsos.171089)
Supplement: Tiee_etal-Table S2 [file rsos171089supp6.pdf]

variable	$LR\ \chi^2$	*d.f.*	*p*-value
museum	36.70	1	1.38E−9
species	14.77	4	0.0052
month of collection	19.17	11	0.058
collection period	21.59	14	0.088
sex	1.69	1	0.19
age of specimen	0.938	11	0.33
